# The Influence of Ketogenic Diet on Gut Microbiota: Potential Benefits, Risks and Indications

**DOI:** 10.3390/nu15173680

**Published:** 2023-08-22

**Authors:** Andrea Santangelo, Antonio Corsello, Giulia Carla Immacolata Spolidoro, Chiara Maria Trovato, Carlo Agostoni, Alessandro Orsini, Gregorio Paolo Milani, Diego Giampietro Peroni

**Affiliations:** 1 Department of Pediatrics, Santa Chiara Hospital, Azienda Ospedaliero Universitaria Pisana, 56126 Pisa, Italy; androsantangelo@gmail.com (A.S.); aorsini.md@gmail.com (A.O.); diego.peroni@unipi.it (D.G.P.); 2 Department of Clinical Sciences and Community Health, Università degli Studi di Milano, 20122 Milan, Italy; giulia.spolidoro@unimi.it (G.C.I.S.); carlo.agostoni@unimi.it (C.A.); milani.gregoriop@gmail.com (G.P.M.); 3 Hepatology Gastroenterology and Nutrition Unit, Bambino Gesù Children Hospital, 00165 Rome, Italy; chiaramaria.trovato@opbg.net; 4 Pediatric Unit, Fondazione IRCCS Ca’ Granda Ospedale Maggiore Policlinico, 20122 Milan, Italy

**Keywords:** ketogenic diet, gut microbiota, children microbiome, gut–brain axis, obesity treatment, drug-resistant epilepsy, ketones and cancer

## Abstract

The ketogenic diet (KD) restricts carbohydrate consumption, leading to an increase in ketone bodies, such as acetoacetate, β-hydroxybutyrate, and acetone, which are utilized as energy substrates. This dietary approach impacts several biochemical processes, resulting in improved clinical management of various disorders, particularly in childhood. However, the exact mechanisms underlying the efficacy of KD remain unclear. Interestingly, KD may also impact the gut microbiota, which plays a pivotal role in metabolism, nutrition, and the development of the immune and nervous systems. KD has gained popularity for its potential benefits in weight loss, blood sugar control, and certain neurological conditions. This narrative review sums up KD-related studies published over 30 years. While short-term studies have provided valuable insights into the effects of KD on the gut microbiota, persistent uncertainties surround its long-term efficacy and potential for inducing dysbiosis. The significant influence of KD on epigenetic mechanisms, intracellular pathways, and gut microbial composition underscores its potential as a therapeutic choice. However, a judicious consideration of the potential risks associated with the strict adherence to a low-carbohydrate, high-fat, and high-protein regimen over prolonged periods is imperative. As KDs gain popularity among the adolescent and young adult demographic for weight management, it becomes imperative to undertake additional research to comprehensively assess their impact on nutritional status and gut microbiota, ensuring a holistic and sustainable approach to medical nutrition.

## 1. Introduction

The ketogenic diet (KD) is a therapeutic dietary approach that is characterized by its low-calorie content, minimal carbohydrate intake, high fat consumption, and standard protein levels. This regimen effectively sustains a persistent state of ketosis, akin to the metabolic state induced by fasting. By restricting carbohydrate intake, KD leads to reduced glucose availability. In response, the liver increases the production of ketone bodies (acetone, acetoacetate, beta-hydroxybutyrate) from fatty acids, as substrates for energy [1]. This approach elicits biochemical processes that have been shown to improve the management of several diseases, also in pediatric age groups [2,3,4,5].

Most common KDs generally restrict the overall consumption of carbohydrates to less than 10% of the total calorie intake, which can be achieved by limiting the intake of carbohydrates to foods with a low glycemic index, typically less than 20–50 g per day [6,7,8]. The Mediterranean diet emphasizes a caloric intake divided into 55% to 60% carbohydrates, 15% to 20% proteins, and 20% to 25% fats, whereas the classic KD involves a significantly low carbohydrate intake, constituting only about 5% to 10% of total calories, along with moderate protein intake ranging from 25% to 35%, and a high fat intake, accounting for about 65% to 70% of the total calories.

Another version of the KD includes a ketogenic diet with medium-chain triglyceride (MCT) supplementation. This variation increases the proportion of medium-chain fatty acids in the diet. Moreover, the modified Atkins diet (MAD) consists of a less restrictive alternative to the classic ketogenic diet. It does not require precise ratio calculations like the traditional KD. MAD allows for slightly higher carbohydrate intake, making it easier to follow while still promoting ketosis.

Finally, the very low-calorie Ketogenic diet (VLCKD) represents an extremely restrictive protocol used for short periods, usually limited to 12 weeks, associated with an overall calorie intake of less than 800 kcal per day. However, the total calorie intake per day is generally unrestricted in pediatric age groups [4,8].

KD could present some side effects, which encompass hypoglycemia, dyslipidemia, gastrointestinal symptoms, bone diseases, nephrolithiasis, and growth failure. Such complications might occur especially at the beginning of the treatment and are easily manageable. However, caution is essential due to potential complications like pancreatitis, cardiac abnormalities, and vascular changes. For such reasons, a qualified nutritionist is needed throughout the treatment. KD is contraindicated in conditions affecting fat metabolism enzymes, porphyria, liver failure, or chronic pancreatitis.

In recent years, these diets have extended beyond their disease-targeted specificity and are frequently used for rapid weight loss purposes [9,10].

The term “microbiota” refers to the different microbial subsets harbored in several tissues within the organism. The gut microbiota is responsible for metabolism, nutrition, and development of the immune and nervous systems. On the other hand, gut dysbiosis has been linked to several pathologies, such as irritable bowel syndrome, asthma, and neurologic or psychiatric disorders [11,12,13]. The gut microbiota seems to have a profound relationship with the central nervous system, jointly shaping the so-called “gut–brain axis”, which has been proven to play a key role in the onset of several neurological diseases [14,15]. Interestingly, different conditions can impact the gut microbiota, such as the environment, pre- and probiotics, drugs, or diet. Modifying these factors could therefore influence the onset, clinical course, and outcome of various disorders, especially in children, where the microbiota is more susceptible to change [16]. However, the impact of KD on the microbiota is still poorly understood, and it could represent a crucial feature in the effectiveness of this treatment.

The purpose of this review is therefore to analyze the current literature in order to recap the possible indications of a KD in pediatric age groups and attempt to provide a better comprehension of its impact on the gut microbiota, which could lead to new approaches to disease prevention and provide a better under-standing of gut–brain axis mechanisms.

## 2. Materials and Methods

This narrative review was conducted through a systematic search of relevant literature using multiple electronic databases, including Pubmed/Medline, Embase, and Web of Science. The review aimed to comprehensively identify and analyze original research papers, meta-analyses, clinical trials, and reviews related to KD published in English within the past 30 years, from May 1993 to May 2023.

To ensure a comprehensive search, three authors independently conducted a literature review and identified the most relevant studies that provided insights into the association between KD and gut microbiota. Various study designs were considered, including systematic and narrative reviews, preclinical and clinical trials, as well as observational studies, covering data from in vitro, in vivo, and human studies. The focus was also extended to pathogenetic mechanisms, including preclinical studies.

The search strategy involved the use of specific keywords related to the ketogenic diet and gut microbiota. Keywords were used alone or in combination to retrieve relevant literature. The primary keywords included “ketogenic diet”, “microbiota”, “bone health”, “pediatric indications”, “children”, and “ketone bodies”. A particular emphasis was placed on studies conducted on infants, children, and adolescents to address the specific implications of the ketogenic diet on gut microbiota and pediatric health. Data from the selected studies were extracted based on their relevance to the topic and analyzed to provide a comprehensive overview of the current understanding of the relationship between KD and gut microbiota in pediatric age groups.

## 3. Indications of KD in Pediatric Age Groups

### 3.1. Epilepsy

Since the beginning of the last century, the KD has been widely adopted as a treatment for epilepsy and is still a recommended therapeutic option for refractory epilepsy in pediatric age groups [17]. However, the specific mechanisms that lead to better seizure control with the KD are not yet fully understood, and several hypotheses have been proposed [18]. 

Various authors have suggested that the KD may affect neurotransmitter levels involved in seizure onset. The activity of the inhibitory neurotransmitter gamma-aminobutyric acid (GABA), for instance, might be enhanced in patients who undergo a KD, both through the activation of glutamic acid decarboxylase and by inhibiting transaminase activity. It has been supposed that the glycolytic adenosine triphosphate (ATP) production enhanced by the KD, and the reduced brain glucose utilization, induce ATP-sensitive potassium channels, increasing the epileptic threshold and then reducing seizures, as has been found in mouse models [19,20]. Therefore, the KD-induced elevation of blood ketone bodies and fatty acids could potentially modulate neuronal membrane excitability through the activation of two-pore domain potassium channels [21]. This mechanism represents a plausible anticonvulsant effect of the KD.

Interestingly, different authors have hypothesized that the KD could also exert a neuroprotective action by upregulating calbindin, inhibiting apoptotic factors such as caspase 3, and increasing the concentration of kynurenic acid [22]. Nonetheless, the KD has been found to reduce oxygen free radicals through the increase of polyunsaturated fatty acids and neuronal uncoupled proteins [23]. All these mechanisms suggest that KD might improve clinical conditions of patients with epilepsy by acting on different biochemical pathways.

To date, different trials have proven the KD to be particularly effective in epilepsy syndromes, leading, in some cases, to the discontinuation of anti-seizure medications. Patients with glucose transporter 1 (GLUT1) deficiency syndrome have shown one of the highest response rates to the KD [24,25]. A study conducted by Kass et al. on 92 children found a seizure reduction greater than 50% in 92% of cases after the initiation of the KD, and 80% of them experienced a seizure reduction of over 90%. Surprisingly, two-thirds of them were not treated with anti-epileptic drugs [26].

Studies have shown both the safety and efficacy of the KD in children suffering from Doose syndrome (epilepsy with myoclonic-atonic seizures). In particular, a multicenter study led by Nickels et al. found at least a 50% reduction in seizures in 79% of patients with Doose syndrome who underwent the KD [27]. Moreover, other trials have proven the KD to be particularly effective in different syndromes, including infantile spasm, pyruvate dehydrogenase deficiency, and tuberous sclerosis complex [28,29,30].

### 3.2. Obesity

During the past decades, obesity prevalence has increased among children, reaching almost 20–30% in several countries [31,32]. Such epidemiologic changes have led to the development of different diet programs, including KD. Despite the concern that a low-carbohydrate, high-fat, and high-protein diet might be unhealthy for obese children, several trial have proven its efficacy in obesity treatment [33,34,35]. 

In fact, different meta-analyses reported in the literature have shown the benefits in terms of weight loss of a low-carbohydrate diet compared to a low-fat one [36]. Nonetheless, reducing carbohydrate intake has also proven to lead to a greater reduction of triglycerides, cholesterol, and diastolic blood pressure levels, while increasing HDL [33]. Such benefits could have been achieved through different mechanisms promoted by KD, including an appetite reduction related to higher levels of glucagon-like peptide 1, cholecystokinin, and ghrelin (so-called “satiety” hormones), or perhaps caused by the suppression of the appetite promoted by ketones themselves [9,10].

Some authors hypothesized that the outcomes of KD in obese patients could also be linked to its efficacy in fat metabolism, since it promotes a higher consumption of fats and an increased lipolysis while reducing lipogenesis [37]. Nonetheless, KD enhances gluconeogenesis, which then leads to a greater energy consumption rate [38]. However, there is still little evidence available to confirm these data and hypotheses among children.

### 3.3. Cancer

Despite its relatively rare incidence (400,000 children and adolescents from 0–19 years-old per year worldwide), cancer represents a leading cause of death and a major concern in pediatric age groups [39]. However, different efforts made by researchers worldwide have led to surprising results: the 10-year survival rate for leukemia in children increased from 27% to 81% in the past 30 years [40]. Nonetheless, due to the lack of treatments available, different therapeutic strategies could be adopted, and KD may represent a useful tool.

By reducing glucose levels, KDs limit the metabolism of cancer cells, which cannot instead process ketone bodies [41]. Such deprivation could also impair glucose-dependent signaling. Moreover, low glucose levels induced by KD lead to a suppression of the lactate/pyruvate cycle, which in turn blocks neovascularization, activation of epidermal growth factor induced by hypoxia, and angiogenesis [42]. Finally, increased levels of ketone bodies could inhibit NLRP3 inflammasome, therefore limiting inflammation, which plays a pivotal role in cancer pathogenesis [43]. 

Different studies have enlightened the potential effect of KD in enhancing the efficacy of chemotherapy and radiotherapy treatments in several malignancies. Neuroblastoma represents a major concern for pediatricians, being the most common extracranial solid malignant tumor diagnosed during the first two years of life, and the most common cancer in children younger than 12 months. Interestingly, a preclinical study in a mouse model by Morscher et al. showed an enhanced anti-neoplastic effect in neuroblastoma, obtained with a combination of cyclophosphamide and KD [44]. 

Acute leukemia represents the most common cancer in pediatric age groups, accounting for 30% of all malignancies. By reducing glycaemia and insulin-secretory response, involved in intratumoral mTORC1 signaling, KD improves the efficacy of anti-PI3K treatment, which could be employed in acute myeloid leukemia [45].

To our knowledge, there are no clinical trials involving pediatric patients assessing the effectiveness of KDs in cancer. However, an interesting case report by Nebeling et al. described the Positron Emission Tomography findings of two girls with malignant astrocytoma following KD for eight weeks, which displayed a decrease of 21.8% in glucose intake at the tumor site [46]. Different studies have also suggested the potential role of KD in treatment of colorectal cancer, glioblastoma, breast, bladder, and pancreatic cancer [41,47,48,49,50]. Despite the promising results, further research is needed on the role of KD in the therapeutic approach to cancer.

### 3.4. Asthma

The possible use of a KD as a targeted therapy for unresponsive asthma has yet to be explored, but possible anti-inflammatory effects of ketones have been already proven on animals. β-hydroxybutyrate, one of the principal ketones in humans, has been found to be a possible endogenous histone deacetylase inhibitor, with a subsequent protective role against oxidative stress [51]. A preclinical study conducted on mice found that increased β-hydroxybutyrate levels reduced the endoplasmic reticulum Ca^2+^ concentrations, which are directly linked to pathways that induce an increased expression of chemokines and metalloproteases in the lung epithelium, which, in turn, are involved in the pathogenesis of asthma [52]. 

Roduit et al. have enlightened that the highest levels of butyrate and propionate in the feces of one-year-old children were associated with a lower risk of developing asthma between 3 and 6 years, suggesting that diet strategies which aim to increase butyrate levels could prevent allergic diseases in children [53].

## 4. Safety and Contraindications

KD is not free from side effects. However, the majority of them are easily manageable. A strict follow-up should therefore be performed, especially in the first few weeks of administration. Common adverse effects may include hypoglycemia, dyslipidemia, gastrointestinal symptoms, carnitine deficiency, bone diseases such as osteopenia and osteoporosis, nephrolithiasis, and even growth failure, and a strict follow-up is then advisable, especially in pediatric patients [54,55,56,57,58,59,60,61]. Some authors have occasionally reported pancreatitis (generally developed in children with more than one risk factor), cardiac abnormalities with prolonged QT intervals, and vascular changes, such as a transient reduction in carotid distensibility [62,63,64,65].

Long-term follow-up studies on epileptic children undergoing a KD have revealed concerning implications for bone health. Increased incidence of bone fractures and decreased bone mineral density have been observed in these patients [66,67,68]. The underlying reasons for these findings warrant careful evaluation. Chronic ketoacidosis, induced by the diet, places heightened demands on bone minerals for buffering capacity and reduces renal conversion of 25(OH)D to 1,25(OH)D [69]. Furthermore, elevated urinary calcium–creatinine ratios have been detected in the absence of hypercalcemia, possibly contributing to kidney stone formation, which occurs in approximately 1 out of 20 patients on the KD [58,70]. Bone mass density (BMD) has also been found to decrease during KD, although identifying patients most at risk of osteopenia remains a challenge. Studies suggest that ambulant children may experience a more significant decline in BMD compared to non-ambulant children [67]. Encouragingly, intravenous bisphosphonate therapy has demonstrated a positive effect in this subgroup [68]. Given these findings, a carefully designed protocol for initiating therapeutic KD and ongoing monitoring of BMD, accompanied by abdominal ultrasound for kidney stones, is crucial. Prophylactic prescription of calcium, vitamin D, and oral potassium citrate has been suggested [63]. It is important to acknowledge that current studies on bone health and KD have been limited to children with epilepsy. Considering that many antiepileptic drugs (AEDs) can also negatively impact bone health and induce bone loss, a comprehensive approach to assessing bone density in children who have followed a KD for an extended duration is essential [54]. AEDs can affect bone health both directly and indirectly [71]. Some AEDs can reduce vitamin D levels, impair calcium absorption, and increase bone turnover, rendering bones more susceptible to fractures. To mitigate these concerns, close monitoring of bone density and potential interventions, such as calcium and vitamin D supplementation, weight-bearing exercises, and lifestyle modifications, should be implemented to minimize the risk of bone loss and fractures in children following long-term KD therapy, strictly monitoring the risk of growth retardation [69].

The administration and adherence to KD must be therefore carefully followed by a pediatrician and a qualified nutritionist. Patients should monitor capillary ketones daily, at least during the initial months, and undergo periodic blood tests, including complete blood count, lipid profile, blood glucose and phosphorus and calcium metabolism, as well as abdominal ultrasound and ECG.

Moreover, contraindications to all forms of KD include altered function of enzymes involved in fat metabolism, including primary carnitine deficiency, carnitine palmitoyl transferase deficiency, carnitine translocase deficiency, or pyruvate kinase deficiency. Porphyria, liver failure or chronic pancreatitis represent other contraindications to this therapeutic option [63].

## 5. Gut Microbiota and Gut–Brain Axis

The gut microbiota is a composite and dynamic environment of bacteria, fungi, viruses, and archea. Together with its metabolites, the gut microbiota plays an important role in human physiology by regulating many essential functions for the host homeostasis, including metabolic, endocrine, nutritional, immune, and neuro functions [72]. 

Studies have shown that the gut microbiota plays a pivotal role in human health and diseases; therefore, any change occurring to the gut microbiota may positively or negatively impact the body homeostasis [73,74,75]. On the other hand, changes in body homeostasis can, in turn, have an impact on gut microbiota. In other words, the relation between the gut microbiota and human body function is not unidirectional, but it is rather a two-way host–microbiome communication system [76]. Among all host–microbiome pathways, the gut–brain axis is the integrated communication system which incorporates together gut and brain functions and acts as a mediator of complex neural processes, such as neural development, neuroinflammation, and neurotransmission [12]. A disruption in the gut–brain axis network can therefore lead to a dysfunction of the axis and, ultimately, to illness. Indeed, many studies have proven a connection between gut dysbiosis and neurological diseases, such as Alzheimer disease [77]. On the other hand, other studies have shown how mental illness can disrupt the gut microbiome homeostasis [72].

Acting on the gut microbiota composition may therefore represent a valid treatment strategy for various illnesses. Other than by administering pre- or probiotics, changes in the gut microbiota composition, structure, and function can be obtained via different dietary regimens, one of which is the keto diet [75]. Indeed, various studies have observed gut microbiota changes in subjects undergoing KD, which could in part explain why KD can be a valid therapeutic approach to some metabolic and inflammatory diseases. However, the mechanisms which could explain how gut microbiome changes may improve illnesses such as epilepsy, obesity, cancer, and asthma are yet to be investigated [78,79,80].

In general, most studies observed that KD alters the microbiome diversity, and consequently, gut metabolites production. These changes in the gut microbiota metabolism appear to have an impact on the communication pathway between gut and brain, and potentially on the whole body. In this light, some studies have proposed that changes in the expression of short chain fatty acid (SCFA), which are gut metabolites that can pass the blood–brain barrier, may explain the modulatory role of KD for certain diseases, although the modalities according to which SCFA may modulate the expression of diseases have not yet been fully clarified [78,79].

## 6. Bone Health and Gut Microbiota

Recent evidence suggests a potential convergence between the gut microbiota, KD, and bone/mineral metabolic complications. The gut microbiome has been proposed as a critical regulator of bone health during postnatal skeletal development and skeletal involution. Abnormal microbiota composition and host responses have been hypothesized to contribute to pathological bone loss [81,82,83]. The probiotic Lactobacillus rhamnosus GG (LGG) has shown promise in increasing bone mass in mice through the production of butyrate, a precursor of β-hydroxybutyrate. LGG or butyrate has been linked to the expansion of T-regulatory cells (T-reg) in both the gut and bone marrow, leading to the upregulation of osteogenic stimulus mediated by CD8+ T-cells. Wnt10b may promote bone formation by activating *Wnt* signaling in osteoblasts [84,85]. Interestingly, SCFAs, including butyrate, have been associated with T-reg and CD8+ T-cell-mediated bone anabolic activity, although SCFAs’ antiresorptive activity may be T cell-independent [81,86]. Additionally, in mouse models with primary and secondary hyperparathyroidism, the presence of segmented filamentous bacteria in the gut microbiota has been implicated in PTH-dependent expansion of intestinal lymphocytes, leading to bone loss [84].

In addition to this possible interaction, it is worth considering the implications of gut-derived serotonin on bone metabolism. Osteoporosis and bone fractures occur more frequently in patients with inflammatory bowel disease, with a decreased BMD [87]. Notably, colitis is characterized by increased serotonin availability in the intestinal mucosa, and gut-derived serotonin has been implicated in reducing bone mass through the activation of serotonin 5-HT1B receptors on pre-osteoblasts [88]. Interestingly, serotonin is produced not only in the gut and the brain but also by certain bacteria, as evidenced by the presence of eukaryote-like aromatic amino acid hydroxylase and aromatic amino acid decarboxylase in multiple bacterial species. This suggests that the human gut microbiota could serve as a potential source of serotonin, playing a crucial role in normal gut physiology and exerting effects on serotonin, serotonin reuptake transporter function, and inflammatory processes [89]. However, further research is needed to fully elucidate the role of gut-derived mucosal serotonin in bone deficits, and how it may interact with other factors, including KD-induced changes in the gut microbiota.

## 7. KD, Epilepsy, and Microbiota: Hypotheses and Evidence

As previously mentioned, a KD may have multiple effects on the human body, both in metabolic and immune function. In addition, like any other long-term diet that significantly alters the balance between different macronutrients, KD can have a significant impact on gut microbiota [72]. Several studies have investigated how a diet almost completely devoid of carbohydrates can positively or negatively alter microbiota composition [90,91,92,93]. Furthermore, it appears that modulation of colonic bacteria plays a direct role in the antiseizure efficacy of KD on pediatric epileptic patients [94,95,96,97].

KD and β-hydroxybutyrate have also been found to increase mean levels of brain GABA, AMP, and adiponectin, which could reduce oxidative stress and potentially impact adipose tissue indirectly [98,99]. The antiseizure effect of KD may also be mediated by a local and specific effect of GABA in the hippocampus, as has been demonstrated in mice [100].

Children with epilepsy have been found to have alterations in their gut microbiota, which could potentially contribute to the development or severity of seizures [101]. Several studies and reviews have investigated the relationship between epilepsy and microbiota, with a focus on the potential impact of the KD on microbiota [102,103,104,105,106]. While the impact of the KD on gut microbiota in children with epilepsy is still an area of active research, these studies suggest that the diet may have a significant impact on the composition and function of gut bacteria. 

A KD has been associated with and increased quantities of *Bacteroidetes* phylum and a degrowth of the microbial diversity and total bacterial abundance after 6 months, maybe due to the deep and normal connection between bacteria and degradation of complex carbohydrates, in order to produce energy from polysaccharides [33,92]. 

As said, a typical effect of the KD is represented by a reduction in serum glucose and BMI, with concomitant ketosis. These signs have been associated in both mouse and human studies with increased gut populations of beneficial bacteria such as *Akkermansia muciniphila*, *Parabacteroides*, *Escherichia coli*, and *Lactobacillus*, while “pro-inflammatory” bacteria such as *Desulfovibrio* and *Proteobacteria* seemed to be reduced [72,78,107,108,109]. Furthermore, a study that divided patients with seizures in responders and non-responders to a KD has found significantly increased quantities of *Clostridiales*, *Ruminococcaceae*, *Rikenellaceae*, and *Lachnospiraceae* in non-responder subjects when compared to responders [108,110]. Another trial conducted on both mice and humans revealed that a ketogenic diet with a fat to protein/sugars ratio of 4:1 resulted in reduced levels of *Bifidobacterium* [111]. The reduction in *Bifidobacterium* was attributed to the increased production of ketone bodies, primarily β-hydroxybutyrate, which consequently led to lower levels of pro-inflammatory Th17 cells in both intestinal and visceral fat. This finding is significant as insulin resistance and obesity are characterized by low-grade inflammation, and reducing Th17 cells may aid in reversing this process. KDs could then lead to decreased intestinal levels of intestinal pro-inflammatory Th17 cells, thanks to their local effect on the microbiota [111]. However, it should be emphasized that the increased abundance of Bifidobacteria is generally associated with improved human health and they are even utilized as common probiotics [112,113].

**Figure 1:**
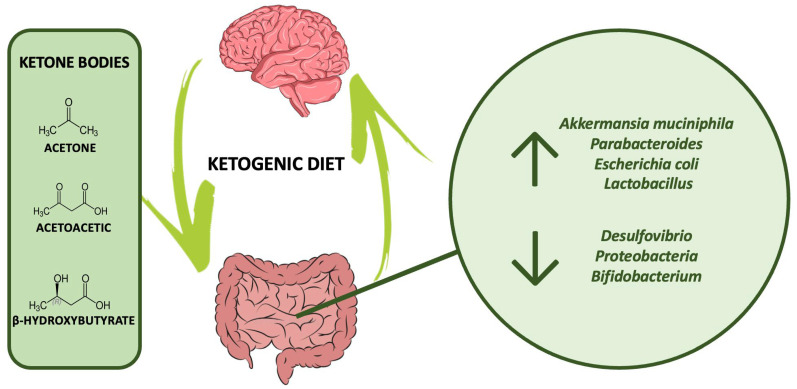
An overview of the possible impact of KD on gut microbiota (increase/decrease).

Figure 1 summarizes the possible impact of a protracted KD on gut microbiota.

Another confirmation of this possible impact of gut microbiota on clinical symptoms of epileptic patients has been shown in studies where both mice and patients with seizures significantly worsened their condition after a high-dose antibiotic treatment, while a successive re-colonization of the gut microbiota improved their symptoms [114].

Moreover, short chain fatty acids, principally produced by Firmicutes, Akkermansia, and Lactobacillus, have been found to significantly improve the ketosis in infants on KD [78,107]. Even if its role on the microbiota could seem contradictory and unclear, the negative correlation on α-diversity richness or increase of bacteria such as Escherichia coli, the increase in short chain fatty acids, and the decrease in lactate levels could prove a positive and beneficial effect on the gut homeostasis [115,116]. KD could then influence the brain not only directly through GABA-mediated mechanisms, but even by microbiome metabolites and short chain fatty acids. Despite any stance, then, these data show how KD has direct involvement with and may modulate the colonic microbiota. 

Considering that many modified versions of KDs are now becoming common in the general population, further studies are needed on their long-term safety and possibilities, as well as considering a possible link between eventual KDs performed during pregnancy or lactation, structuring the infant gut microbiota, and the onset or prevention of complex and multifactorial conditions such as epilepsy or asthma [117].

## 8. Conclusions

This review highlights the potential of microbiota plasticity as a promising avenue for disease prevention and treatment, with the KD showing potential in influencing various diseases and underlying mechanisms. Nevertheless, it is essential to acknowledge that the current evidence related to children and the long-term effects of dietary interventions remain limited, necessitating cautious interpretation.

While short-term studies have provided insights into the effects of KD on the gut microbiota, uncertainties persist regarding the long-term efficacy and potential for dysbiosis. The impact of KD on epigenetic mechanisms, intracellular pathways, and gut flora underscores its potential as a therapeutic approach. However, it is crucial to consider the potential risks associated with strict adherence to a low-carbohydrate, high-fat, and high-protein diet, particularly over extended periods. As KDs gain popularity among adolescents and young adults for weight loss, additional research is needed to assess their effects on nutritional status and gut microbiota to ensure a balanced and sustainable approach to medical nutrition.

In conclusion, the findings of this review suggest that exploring the interactions between KD, microbiota, and various diseases can provide valuable insights into novel therapeutic approaches. However, due to the limited evidence related to children and the potential long-term consequences of KD, further research is warranted. A cautious and comprehensive approach to understanding the effects of KD on microbiota, metabolism, and pediatric health is essential to optimize its therapeutic potential and inform dietary recommendations for different populations in the future.

## Data Availability

Not applicable.
